# Magnitude of diabetic peripheral neuropathy in Saudi Arabia: a systematic review and meta-analysis

**DOI:** 10.1186/s12902-022-01167-4

**Published:** 2022-11-02

**Authors:** Lukman Femi Owolabi, Mushabab Alghamdi, Bappa Adamu, Magaji Garba Taura, Abubakar Jibo, Mohammed Almansour, Saeed Naseer Alaklabi, Mohammed Ali Alghamdi, Yousef Ayesh Alotaibi, Isa Adamu Imam, Reda Abdelrazak, Ahmad Rafaat, Muktar Hassan Aliyu

**Affiliations:** 1 University of Bisha Medical College, Bisha, Aseer region Saudi Arabia; 2 King Fahd Medical city, Riyadh, Saudi Arabia; 3 King Abdullah Hospital, Bisha, Anseer region Saudi Arabia; 4 Vanderbilt University, Nashville, USA

**Keywords:** Diabetic peripheral neuropathy, Magnitude, Prevalence, Meta-analysis, Saudi Arabia

## Abstract

**Background:**

Diabetic peripheral neuropathy (DPN), due to its potential for causing morbidity and disability from foot ulcers and amputations, is increasingly becoming a source of concern in Saudi Arabia and worldwide. However, wide variability exists in the prevalence of DPN reported in previous studies in Saudi Arabia, limiting the utility of existing data in national public health policy. Therefore, the aim of this study was to systematically evaluate the magnitude of DPN in patients living with DM in Saudi Arabia in order to inform policymakers during the implementation of appropriate preventive and treatment strategies for DPN.

**Methods:**

PubMed, Google Scholar, African Journals Online, Scopus, Web of Science, Embase, and Wiley Online Library were searched systematically to acquire relevant articles based on preset criteria. We evaluated heterogeneity and publication bias and employed a random-effects model to estimate the pooled prevalence of DPN from the included studies. We followed the Preferred Reporting Items for Systematic Reviews and Meta-Analyses guidelines in conducting the meta-analysis. Analysis was performed using the STATA Version 12 software.

**Results:**

Twelve studies with a total of 4,556 participants living with DM, of whom 2,081 were identified as having DPN were included in the meta-analysis. The overall prevalence of DPN was 39% (95% CI [30%, 49%]). Subgroup analysis based on diagnostic method showed that prevalence estimates for DPN using screening questionnaires and clinical examination were 48% (95% CI [46%, 50%]) and 40% (95% CI: [38%, 42%]), respectively, while the estimated prevalence using nerve conduction studies was 26% (95% CI [15%, 36%]).

**Conclusion:**

This study showed a high magnitude of DPN in Saudi Arabia (39%), thus highlighting the need for sustained efforts to reduce the prevalence of diabetes mellitus and DPN in the kingdom.

## Introduction

Diabetes mellitus (DM) remains a major worldwide health concern, and diabetic peripheral neuropathy (DPN) is the most common cause of peripheral neuropathy globally [1]. According to consensus, DPN is defined as a symmetrical and length-dependent sensorimotor polyneuropathy resulting from alteration in metabolism and small vessels caused by the prolonged effect of hyperglycemia and metabolic abnormalities [2]. Nearly half of those with DM have DPN, and 1–2 out of 10 patients with DPN have severe cases that warrant treatment [3, 4].

DM is undoubtedly one of the most challenging health problems facing Saudi Arabia [5]. The Ministry of Health of Saudi Arabia estimated that the number of people diagnosed with DM rose from approximately 0.9 million people in 1992 to 2.5 million people in 2010, representing an almost 3-fold increase in incidence over less than 2 decades [6]. A recent meta-analysis of observational studies in Saudi Arabia reported type 2 DM, type 1 DM, and overall point prevalence rates of 20.9%, 0.9%, and 12.6%, respectively [7].

DM is associated with morbidity and mortality of public health significance. Complications associated with DM are increasingly a source of concern in several Arab populations [8]. Notable among the numerous complications of DM is DPN, a common microvascular complication with an attendant risk of ulceration and amputation [9]. that accounts for a large economic burden of diabetes care.

Globally, DPN places a large economic burden on both people living with DM and national healthcare systems. The annual cost of treatment for DPN and its related complications in people living with DM in the United States was estimated at US$10.91 billion [10]. The care of diabetic foot, a common complication of DPN, accounted for approximately 0.6% of National Health Service expenditures between 2010 and 2011 [11]. Though there is a paucity of data on the total cost of treating people with DM in Saudi Arabia, the national healthcare burden of diabetes was, without considering indirect costs, estimated to exceed US$0.87 billion [12]. DPN thus contributes significantly to the health expenditures of both Saudi Arabia and the world at large. Therefore, knowledge of the magnitude of DPN to inform policies relating to controlling the substantial health expenditures from DPN is a country-specific as well as a global concern.

Wide variability exists in the prevalence of DPN reported in previous studies. Estimates of the magnitude of DPN include 56.2% in Yemen [13], 48.1% in Sri Lanka [14], 46% in Africa [15], 39.5% in Jordan [16], 29.2% in India [17], 8.4% in China [18], 20% in France [19] and 11–25% in the United States [20, 21]. A systematic review conducted on painful DPN reported a point prevalence of 43.2%, though the review included only 1 study from Saudi Arabia [22].

In Saudi Arabia, data on the prevalence of DPN vary remarkably by study, with reported DPN prevalence rates ranging from 20% [23] to 66.7% [24]. However, most of these reports had small sample sizes, limiting the extent to which they can be used as national figures of the magnitude of DPN. This marked variation in DPN prevalence estimates could be ascribed to heterogeneity across studies in study design, risk factors, population demographics, and case ascertainment, constraining the use of existing data to estimate the number of people in Saudi Arabia with DPN and guide national public health policy.

The aim of this study was thus to systematically evaluate the magnitude of DPN in patients living with DM in Saudi Arabia in order to inform policymakers during the implementation of appropriate preventive and treatment strategies for DPN.

## Materials and methods

### Literature search

We searched the electronic databases PubMed, African Journals OnLine, Scopus, Web of Science, and Embase. Cambridge Middle East Library and Cochrane Reviews were also searched. Furthermore, we performed a hand search of grey literature and other related articles as well as a review of the reference lists of already gathered articles in order to retrieve additional relevant studies. A combination of Medical Subject Heading search terms related to DPN (“diabetic peripheral neuropathy,” “peripheral neuropathy” “diabetic neuropathy,” “diabetic polyneuropathy” “prevalence,” “magnitude,” “Saudi Arabia,” “Kingdom of Saudi Arabia”), employed in combination with Boolean operators such as AND and OR to connect search terms, comprised the search strategy. To reduce potential publication bias, we also searched conference proceedings, technical reports on DPN, and medical organization websites. The search was carried out between July 1, 2020, and January 30, 2021 and updated up to August 31, 2022. The search was carried out in English language. Independent searches were conducted by both the investigators and a librarian.

## Selection criteria and process

### Inclusion criteria

Studies were included if they met the following criteria: (1) the studies had cross-sectional or case-control designs investigating the prevalence or magnitude of DPN, (2) DM and DPN diagnoses were medically confirmed, (3) outcome prevalence or magnitude was reported as the outcome variable or prevalence was not reported but sufficient data was present to compute prevalence of DPN, and (4) the studies were conducted on the Saudi population.

### Exclusion criteria

Studies were excluded based on the following criteria: (1) they provided inadequate or ambiguous information regarding the prevalence or mode of diagnosis of overweight and obesity or (2) they provided prevalence estimates for biased populations, such as populations of pregnant women or prison inmates.

### Screening of studies

We initially screened the titles and abstracts of all gathered articles and then conducted a full-text review to identify articles that were eligible for further review.

### Data extraction

We extracted relevant data for this systematic review using a Microsoft Excel form designed to capture the information of interest from each article.

For each included study, we obtained information regarding the author, year of study, year of publication, study setting, study type, study population, data collection and analysis methods, and mean age of study participants. We used the name of the study’s first author and the year of publication to code the data. After extracting the data independently, the investigators verified all data against the predetermined inclusion and exclusion criteria. Potentially eligible studies were reviewed independently by 2 investigators. Any disagreements were reconciled by discussion or by the third investigator.

### **Reporting format and quality** assessment **and**

**Fig. 1:**
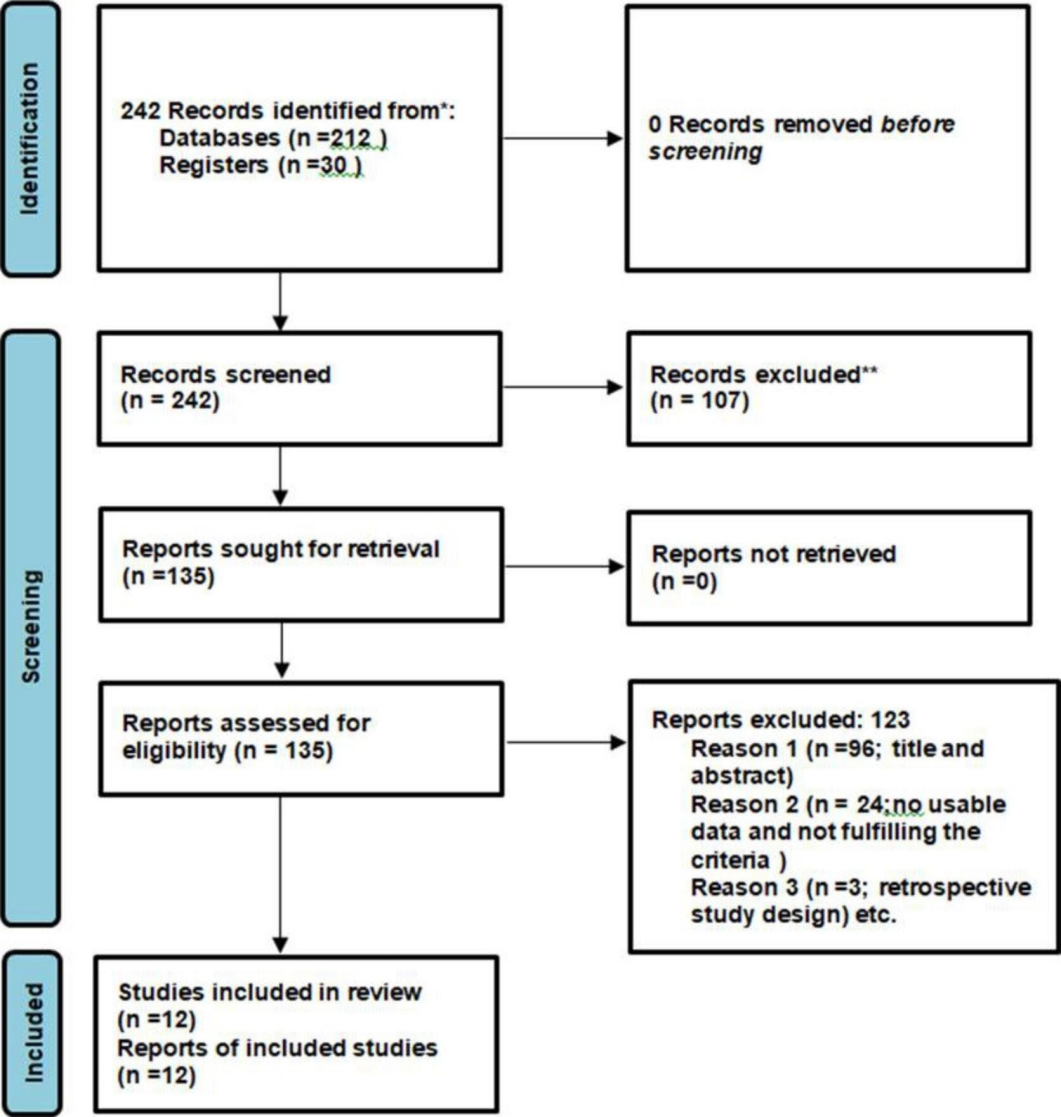
PRISMA Flow diagram showing the process of selection of articles for the meta-analysis

We performed the meta-analysis based on the Meta-analyses of Observational Studies in Epidemiology (MOOSE) [25]. guidelines and the Preferred Reporting Items for Systematic Reviews and Meta-Analyses (PRISMA) statement [26]. (Fig. 1). The Agency for Healthcare Research and Quality (AHRQ) checklist for cross-sectional reports was utilized to assess the methodological quality of the included studies. Two investigators carried out the assessment of study quality and any disagreements at the time of quality scoring were reconciled by discussion and by the third investigator.

### Data analysis

The prevalence (P) estimate of DPN in Saudi Arabia was the outcome of primary interest in this review. For each selected study, the prevalence of DPN was calculated and expressed as percentages with 95% confidence intervals (CIs). A binomial probability distribution was employed to determine the standard error of the prevalence. The log of the prevalence (logP) and the standard error of logP were calculated for all selected studies. Using a random-effects model (REM) proposed by DerSimonian and Laird, summary estimates of DPN prevalence and 95% CIs were generated [27]. We used between-study heterogeneity tests, Cochran’s Q chi-squared statistics, and I^2^ statistics to assess the random variations between studies. Results with I^2^ > 50% were considered to reflect substantial heterogeneity. Potential random variations between the point estimates of the studies were minimized and the sources of heterogeneity observed in the analysis were investigated through meta-regression, subgroup analysis, and sensitivity analysis.

We assessed publication bias and smallstudy effects by visual inspection of the funnel plot. Egger’s regression asymmetry test and Begg’s adjusted rank correlation test were also conducted to identify the presence of publication bias [28, 29]. A p value ≤ 0.05 was considered statistically significant for the presence of publication bias. In order to reduce the inconsistency and insensitivity that may arise from these tests [30], we considered the existence of publication bias if it was determined by both tests.

The meta-analysis was performed using the STATA version 12 statistical software for Windows (Stata Corp., College Station, TX, USA).

## Results

### Study identification and selection

Our search strategy yielded 242 initial unique citations. After the removal of duplicate articles, there were 134 articles that were potentially relevant (Fig. 1). The screening of titles and abstracts based on the exclusion criteria resulted in the removal of 123 articles Finally, 11 cross-sectional studies [8, 24, 31, 32, 33, 34, 35, 36, 37, 38, 39] and 1 prospective study [40]. were assessed for their quantitative analysis (Fig. 1). Three additional studies [41–43]. were removed due to their retrospective design.

The studies that met the predetermined criteria consisted of a total of 4,556 people living with DM, of whom 2,081 were identified as having DPN. These studies were included in the final analysis. The results of the quality assessment based on AHRQ standards are shown in Table 1, while the characteristics of the included studies are presented in Table 2.

**Table 1:** Showed the results of quality assessment based on AHRQ* quality standards applied to included studies

Study Identification	Define source of information	List inclusion and exclusion criteria	Indicate time period used for identifying patients	Indicate whether participants were consecutive if not population-based	Indicate if evaluators of subjective components of the study were masked to other aspects of the status of the participants	Describe Assessment undertaken for quality assurance purposes	Explain any patient exclusions from analysis	Describe how confounding was assessed or controlled	Explain how missing data were handled in the analysis	Total
Nielsen et al. [31]	1	1	1	1	1	1	1	1	0	8
Akbar et al. [32]	1	1	1	1	1	1	1	0	0	7
Mojaddidi et al. [40]	1	1	1	1	1	1	1	0	0	7
Halawa et al. [8]	1	1	1	1	1	1	1	1	0	8
Ahmed et al. [33]	1	1	1	1	1	1	0	0	0	6
Wang et al. [34]	1	1	1	1	1	1	1	1	1	9
Kasim et al. [35]	1	1	1	1	1	1	1	1	0	8
Shesha et al. [36]	1	1	1	1	1	1	1	0	0	7
Shesha et al. [36]	1	1	1	1	1	1	0	1	0	7
Algeffari et al. [24]	1	1	1	1	1	1	0	0	0	6
Aljehani et al. [37]	1	1	1	1	1	1	1	0	0	7
Sendi at al. [38]	1	1	1	1	1	1	1	1	0	8
Almohaysen et al. [39]	1	1	0	1	1	1	1	1	0	7

*AHRQ = Agency for health research quality (Two columns namely; (a)Summarize patient response rates and completeness of data collection and b) Clarify what follow-up, if any, was expected and the percentage of patients for which incomplete data or follow-up was obtained, in which all the studies scored 0 were deleted)

**Table 2:** Showed the characteristics of the included studies

Author	Year of study	Year of publication	Type of study	Mean age	Proportion of female	Region	Type of DM	Screening technique	Total	Cases	Prevalence	Quality/10
Nielson et al. [31]	1996	1998	Cross sectional	50*	38*(of dn pat)	Riyadh	T_2_	Clinical	375	143	38.1	7
Akbar et al. [32]	1999	2000	Cross sectional	54.19	NA	Jeddah	T_1_ + T_2_	MNSI	237	132	55.7	6
								NCS	237	58	24.4	
Mojaddidi et al. [40]	2009	2011	Prospective	51.79	54.3	Al-Madinah	T_1_ + T_2_	DNS + DNI	263	165	62.7	9
								NCS	263	43	16.4	
Halawa et al. [8]	2007	2010	Cross sectional	52.10	33.4	Multicentre	T_1_ + T_2_	DN4	1039	619	59.6	9
Ahmed et al. [33]	2012	2014	Cross sectional	51.43	43.0	Jeddah	T_2_	Standardized assesment form	350	168	48.0	5
Wang et al. [34]	2010	2014	Cross sectional	59.50	38.2	Jeddah	T_1_ + T_2_	Clinical	552	110	19.9	8
Kasim et al. [35]	2012	2015	Cross sectional	51.50	58.0	Al-Madinah	T_1_ + T_2_	MDNS	100	20	20.0	7
Shesha et al. [36]	2013	2017	Cross sectional	45.40	41.5	Riyadh	T_2_	NSS/NDS	125	44	35.2	8
								NCS	87	34	39.1	
Algeffari et al. [24]	2013	2018	Cross sectional	56.90	66.7	Riyadh	T_2_	MNSI	242	84	34.7	8
Aljehani [37]	2019	2019	Cross sectional	50.62	62.7	Riyadh	T_1_ + T_2_	Clinical	469	324	69.1	8
Sendi at al. [38]	2019*	2020	Cross sectional	NA	54.7	Al-Madinah	T_1_ + T_2_	DN4	430	129	30.0	7
Almohaysen et al. [39]	NA	2020	Cross sectional	53.61	54.0	Al-Qassim	T_2_	MDNS	374	143	38.2	8

*T_1_:Type-1DM, T_2_:Type-2DM,NCS: Nerve conduction study, MNSI: Michigan Neuropathy Screening Instrument, MDNS: Michigan Diabetic Neuropathy Score, DN4: DN4 assessment tool, NSS: Neurological symptom score, NDS: Neuropathy Disability Score

### Risk of bias assessment

As retrospective studies found in our literature searches were not considered for the final analysis, the included studies were cross-sectional and prospective (cohort) studies, with no case-control studies. Three [32, 36, 40]. of the cross-sectional studies were used in the final analysis because they used 2 diagnostic methods (questionnaire-based and nerve conduction studies) and arrived at 2 different prevalence estimates for the same population.

### Overall prevalence of diabetic peripheral neuropathy in Saudi Arabia

**Fig. 2:**
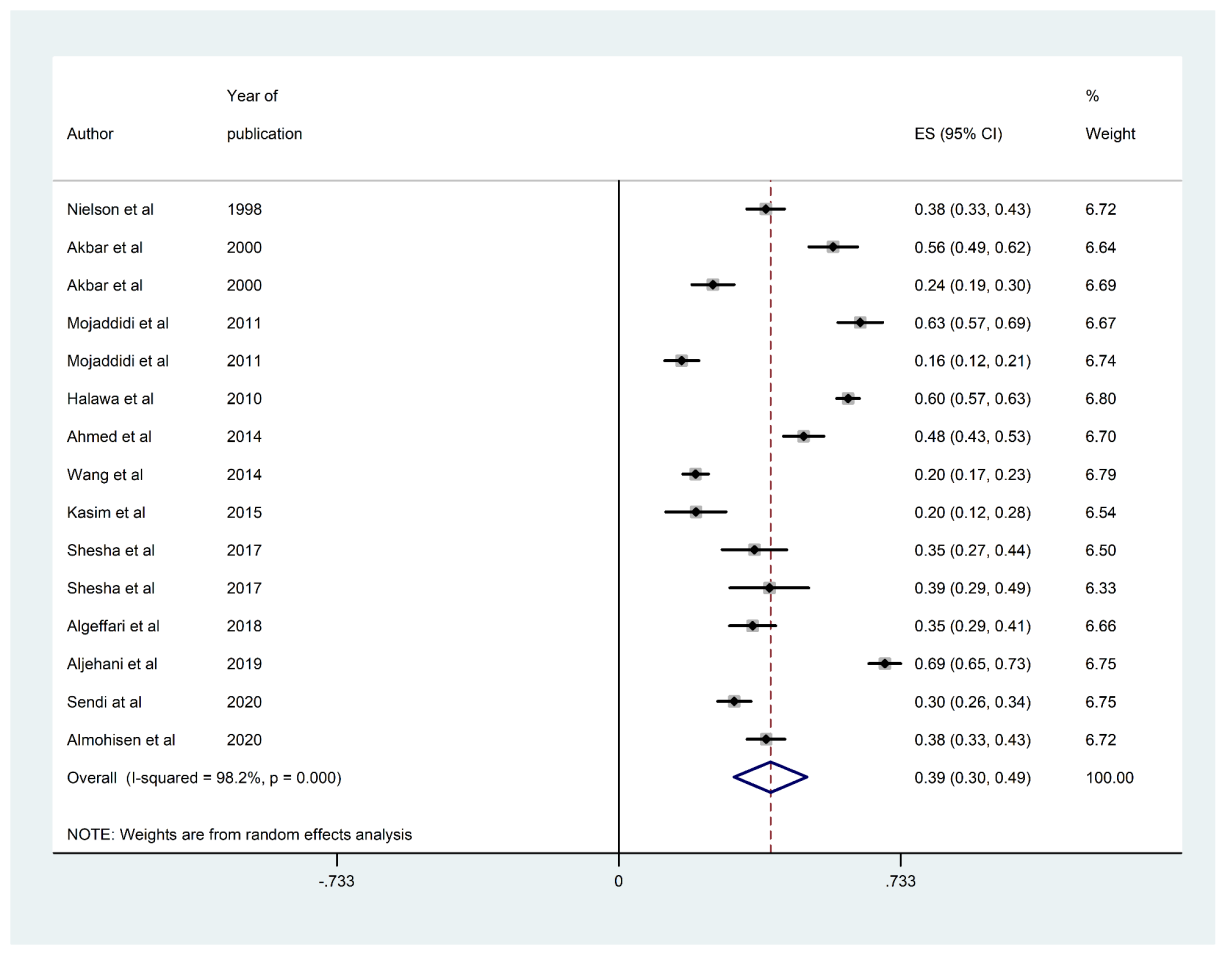
Forest plot of studies included in meta-analysis with pooled prevalence of DPN (39%) in Saudi Arabia

**Fig. 3:**
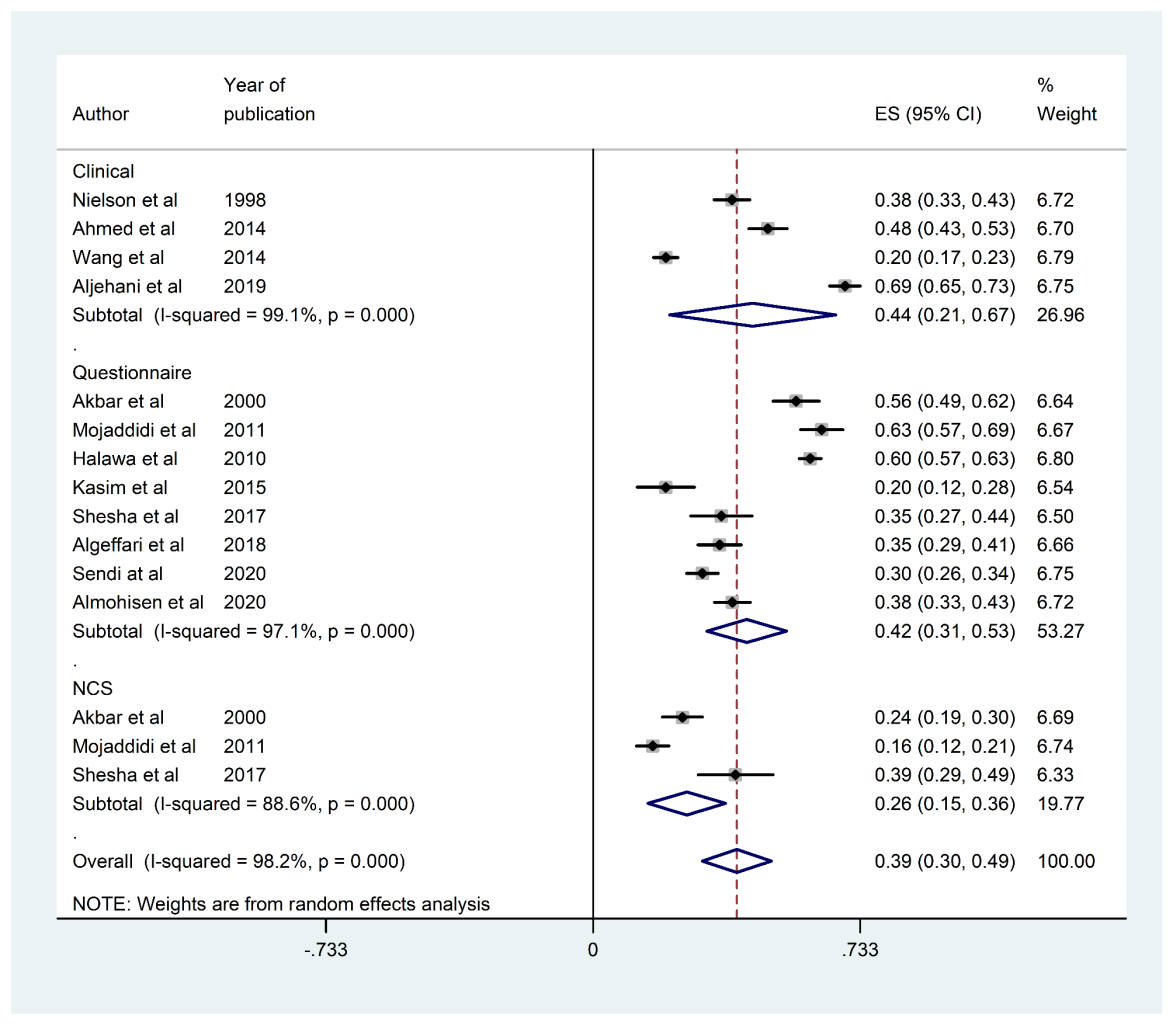
Funnel plots showing graphic representation of Publication bias with Funnel plot of the included studies (A) and using Trim and Fill method (B)

The overall estimated prevalence of DPN in Saudi Arabia based on the included studies was 39% (95% CI [30%, 49%]; Fig. 2). The I^2^ test revealed marked heterogeneity (I^2^ = 98.2% and p < 0.001). Egger’s test for small-study effects showed evidence of publication bias (p < 0.0001). Begg’s test also showed publication bias (p < 0.0001). In agreement with these results, the funnel plot was asymmetric even after adjusting for potential publication bias by applying the trim-and-fill method (Fig. 3).

### Publication bias

Publication bias was assessed using Egger’s test for small-study effects. There was no evidence of publication bias (p < 0.0001). Begg’s test also showed publication bias (p = 0.0001). In agreement with these findings, the funnel plot was asymmetric even after adjusting for potential publication bias by applying the trim-and-fill method (Fig. 3).

## Sources of heterogeneity: subgroup and regression analyses

### Subgroup analysis

**Fig. 4:**
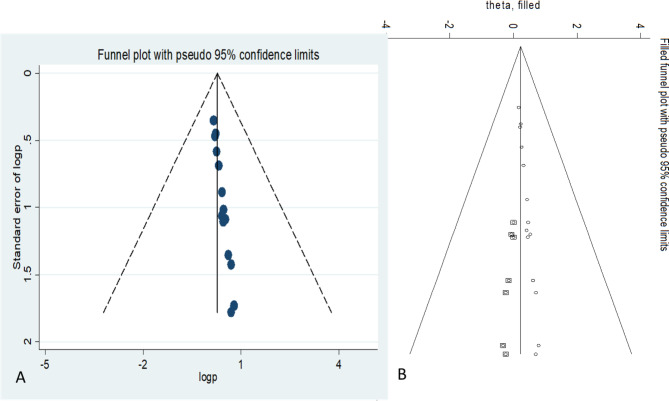
Forest plot showing subgroup prevalence estimates on method of diagnosis

Due to the marked heterogeneity observed, which can be ascribed to different estimate effect modifiers such as the diagnostic method employed in the individual studies, we performed subgroup analyses using these variables. Subgroup analysis found that the estimated DPN prevalence rates using screening questionnaires and clinical examination were 48% (95% CI [46%, 50%]) and 40% (95% CI [38%, 42%]), respectively, while the estimated prevalence using a nerve conduction study was 26% (95% CI [15%, 36%]; Fig. 4). The I^2^ test revealed marked heterogeneity (I^2^ = 98.2% and 88.6%, respectively, p < 0.001).

### Meta-regression analysis

**Fig. 5:**
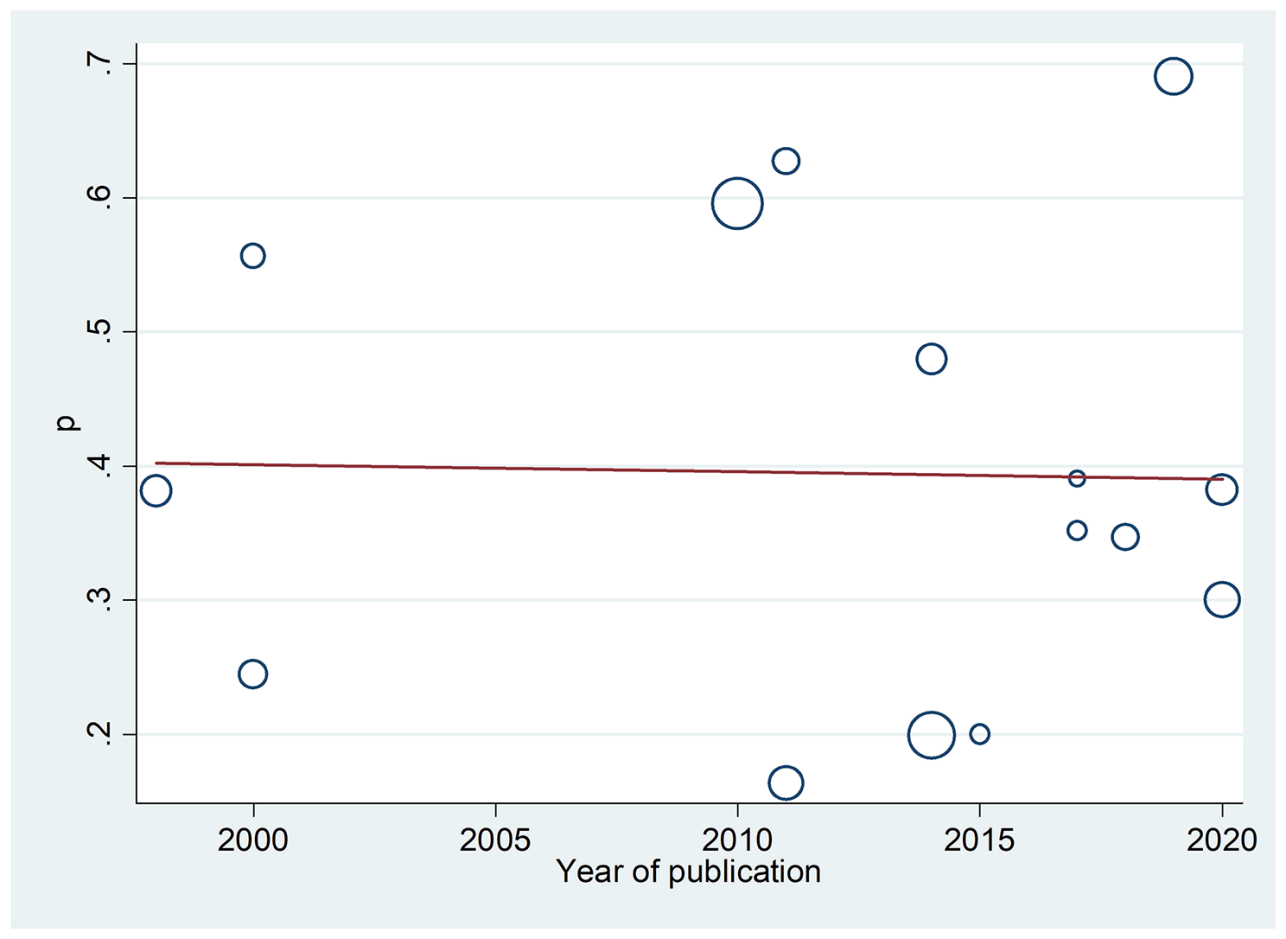
Meta-regression plot showing the trend in DPN prevalence over the years

We further investigated the possible sources of between-study variation in our analysis by performing meta-regression analysis of sample size against publication year as variables of interest. The result of the meta-regression analysis showed that neither covariate was significantly associated with the presence of heterogeneity (− 0.0006, 95% CI [− 0.014, 0.0129], p = 0.931; Fig. 5).

### Sensitivity analysis

**Fig. 6:**
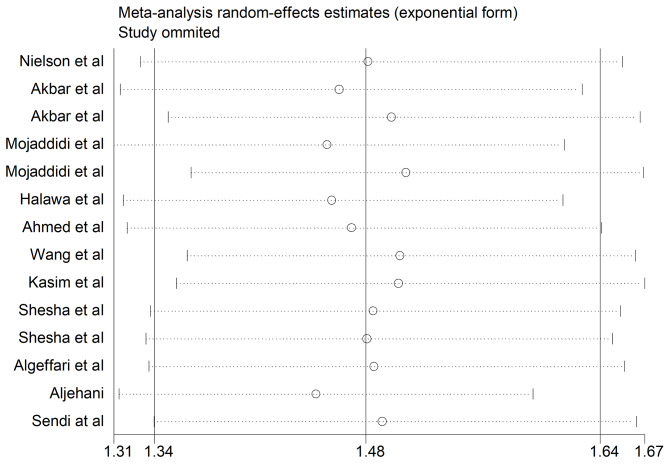
Sensitivity analysis showing no significant influence of any of the included studies on the others

We carried out sensitivity analysis to evaluate the impact of each study on the pooled estimate of DPN prevalence. Using a random-effects model, our analysis showed that no single study influenced the overall prevalence of DPN (Fig. 6).

## Discussion

The current study found an overall prevalence of DPN in Saudi Arabia of 39%. The estimated DPN prevalence in Saudi Arabia calculated in this analysis appears to be lower than reports from similar studies conducted in developing countries [15, 44]. Meta-analyses of observational studies in Iran and Africa reported estimated prevalence rates of 53% [44] and 46% [15], respectively. In contrast, our result is higher than the prevalence findings in Oceania, the Americas, and Asia of 23.2%, 31.6%, and 32.24%, respectively [45]. Similarly, the prevalence estimate obtained in the current study is higher than the global prevalence of DPN among people living with type 2 DM of 35.78% [45]. This global variability in DPN prevalence has been ascribed to differences in regional characteristics and potential risk factors, including duration of DM, patient age, patient height, and glycemic control status [45].

Although the pathomechanism of DPN remains largely unclear, it is increasingly recognized as involving a multitude of factors that include vascular occlusion of the vasa nervorum, deficiency of myoinositol with alteration of myelin synthesis, endothelial abnormality, hyperosmolarity with resultant nerve edema and disruption of the nerve architecture, dysfunction of sodium–potassium adenine triphosphatase, and the impacts of the accumulation of fructose and sorbitol [46]. Peripheral nerve dysfunction in people with DPN is largely irreversible, which necessitates increased attention devoted to prevention through the identification of modifiable risk factors for DPN [47]. To this end, previous studies have identified some common risk factors responsible for DPN in people living with DM, including duration of DM, patient age, gender, existence of microvascular complications, alcohol consumption, hypertension, obesity, cigarette smoking, physical inactivity, and glycated hemoglobin level.These risk factors have been found to facilitate development of DPN in patients with DM [17, 47, 48].

The prevalence of DPN reported by the studies included in the current meta-analysis varied widely, ranging from 20% [23] to 66.7% [24]. This discrepancy, which could be explained by heterogeneity in diagnostic method, was further explored by subgroup analysis based on the studies’ method of case ascertainment, consisting of clinical examination (symptoms and signs), screening questionnaires, and electrodiagnostic testing. The primary screening questionnaires used in the studies included the Michigan Neuropathy Screening Instrument (MNSI), Neuropathy Symptom Score (NSS), and Neuropathy Disability Score (NDS). The sensitivity of MNSI (using a cut-off point of 2.0), NSS, and NDS are 65%, 82.05%, and 92.3%, respectively, and their specificities are 83%, 66.67%, and 47.62%, respectively [45, 49].

The diagnostic methods employed differed among the studies due to differences in study aims. The prevalence of DPN based on the method of case ascertainment was highest (48%) with screening questionnaires and lowest (26%) with electrodiagnostic tests.

There is limited evidence indicating an ideal screening method for DPN. Nonetheless, there has been remarkable progress in the detection of DPN in people living with DM with particular reference to electrophysiological techniques and quantitative sensory tests [50].

### Strengths and weaknesses of the study

To the best of our knowledge, this meta-analysis is the first to quantitatively pool data from carefully selected studies to generate estimates of the magnitude of DPN in Saudi Arabia. The present meta-analysis was conducted based on PRISMA guidelines for meta-analyses of observational studies. We carried out a comprehensive search strategy to include studies from Saudi Arabia using stringent predetermined criteria, assessed the quality of the selected studies with a robust critical appraisal tool, employed multiple standardized methods to quantify publication bias in our study, and further explored the heterogeneity observed in the analysis using meta-regression and sensitivity analysis.

Despite the strengths of our study, this meta-analysis also has some limitations that warrant mentioning. First, only studies published in the English language were included in the analysis, introducing the potential to exclude relevant data published in other languages. Nevertheless, the official language of medical journals in Saudi Arabia is English; thus, the possibility of missing relevant non-English medical publications is low. Second, publication bias was found during our analysis. We made an effort to mitigate publication bias by attempting to retrieve related but unpublished work, and we statistically explored this by applying the trim-and-fill technique. It is worth noting that our results do not undervalue the fact that a robust, rigorous, well-designed, and well-conducted national epidemiological survey, performed concurrently across all regions of Saudi Arabia using a consistent methodological approach, would deliver a more reliable magnitude of DPN in Saudi Arabia. In the absence of such an effort, our findings provide robust estimates of the magnitude of DPN in Saudi Arabia and can guide the planning of prevention and treatment strategies for DPN in the kingdom [1, 21, 24].

It is additionally important to note that the heterogeneity of clinical manifestations of DPN makes it difficult to identify patients at high risk for DPN. Therefore, early diagnosis is key to a better prognosis and prevention of diabetic foot ulcers, amputation, and DPN-related disability.

## Conclusion

This study showed that the overall prevalence of DPN in Saudi Arabia was high (39%). Though lower than average prevalence figures in developing countries, there is need to implement sustainable preventive strategies and interventions to stem this preventable complication of DM. Additionally, the need for a sustained effort aimed at identifying associated factors for DPN in patients with DM in Saudi Arabia cannot be overemphasized.

## Data Availability

All data generated or analyzed during this study are included in this published article.
